# Supplementary material for: Molecular epidemiology and antimicrobial resistance phenotype of paediatric bloodstream infections caused by Gram-negative bacteria
Source: Commun Med (Lond). 2022 Aug 11;2:101. doi: 10.1038/s43856-022-00161-0 (PMC9372158; doi:10.1038/s43856-022-00161-0)
Supplement: Supplementary file 1 — Supplementary Information [file 43856_2022_161_MOESM1_ESM.pdf]

Supplementary material for:

Molecular epidemiology and antimicrobial resistance phenotype of paediatric bloodstream infections caused by Gram-negative bacteria in Oxfordshire, UK

Sam Lipworth, Karina-Doris Vihta, Tim Davies, Sarah Wright, Merline Tabirao, Kevin Chau, Alison Vaughan, James Kavanagh, Leanne Barker, Sophie George, Shelley Segal, Stephane Paulus, Lucinda Barrett, Sarah Oakley, Katie Jeffery, Lisa Butcher, Tim Peto, Derrick Crook, Sarah Walker, Seilesh Kadambari* and Nicole Stoesser*

Supplementary Methods

Virulence genes searched for using Kleborate (BLAST hits) in *Klebsiella spp.*

Yersiniabactin (ybt), aerobactin (iuc), salmochelin (iro) and colibactin (clb). Searches were performed using default settings (minimum 90% identity and 80% coverage).

Table S1 – Reference sequences used

Species (Sequence type)	Reference
E. coli (131)	HG941718.1
E. coli (95)	NZ_CP012625.1
E. coli (73)	AE014075.1
E. coli (69)	Custom reference, available at 10.6084/m9.figshare.14785854
E. hormaechei	NZ_CP032841.1
S. marcescens	NZ_KQ089767.1

Table S2: comparison of phenotypes for community-acquired vs. healthcare-onset bloodstream infection isolates. S – Susceptible, R – Resistant, p values represent Fisher's exact tests.

	Phenotype	Community acquired	Healthcare associated	p
Amoxicillin	S	59 (33.9)	19 (16.1)	0.001
	R	115 (66.1)	99 (83.9)	
Ceftriaxone	S	162 (87.6)	99 (83.9)	0.396
	R	23 (12.4)	19 (16.1)	
Ciprofloxacin	S	182 (94.8)	118 (94.4)	1.000
	R	10 (5.2)	7 (5.6)	
Amikacin	S	72 (93.5)	52 (91.2)	0.743
	R	5 (6.5)	5 (8.8)	
Gentamicin	S	178 (92.7)	110 (88.7)	0.231
	R	14 (7.3)	14 (11.3)	
Piperacillin-Tazobactam	S	172 (92.5)	105 (86.8)	0.117
	R	14 (7.5)	16 (13.2)	
Fosfomycin	S	54 (91.5)	45 (91.8)	1.000
	R	5 (8.5)	4 (8.2)	

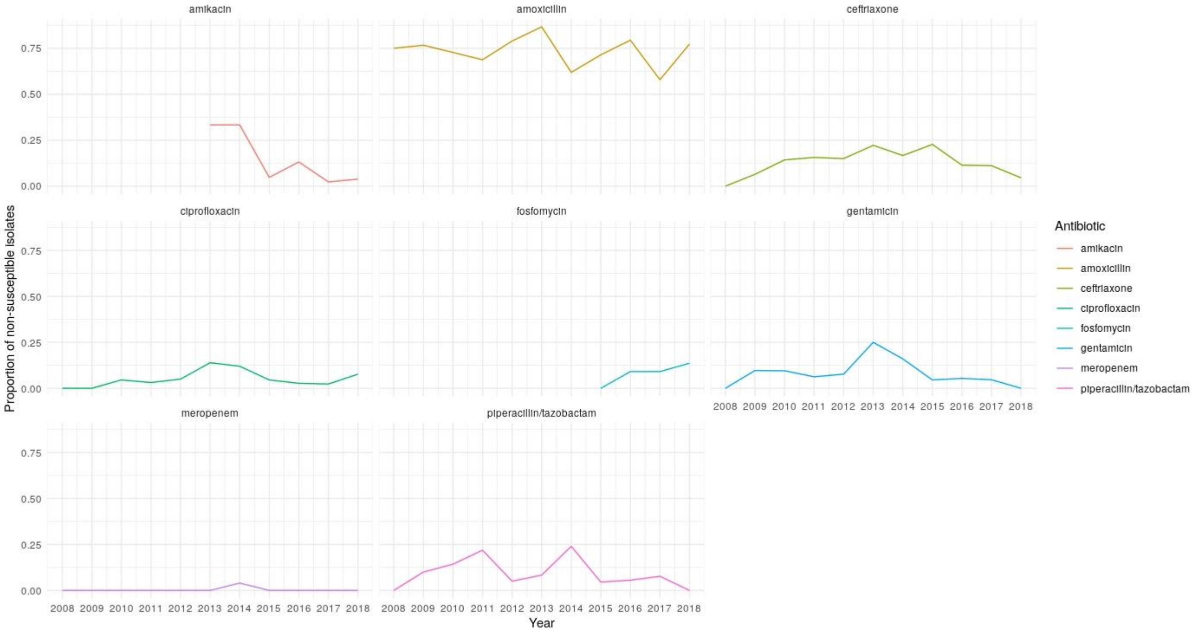

Figure S1: Proportion of non-susceptible isolates over time. Routine testing for amikacin and Fosfomycin (2013 and 2015 respectively) started later, hence the blank areas in these plots.

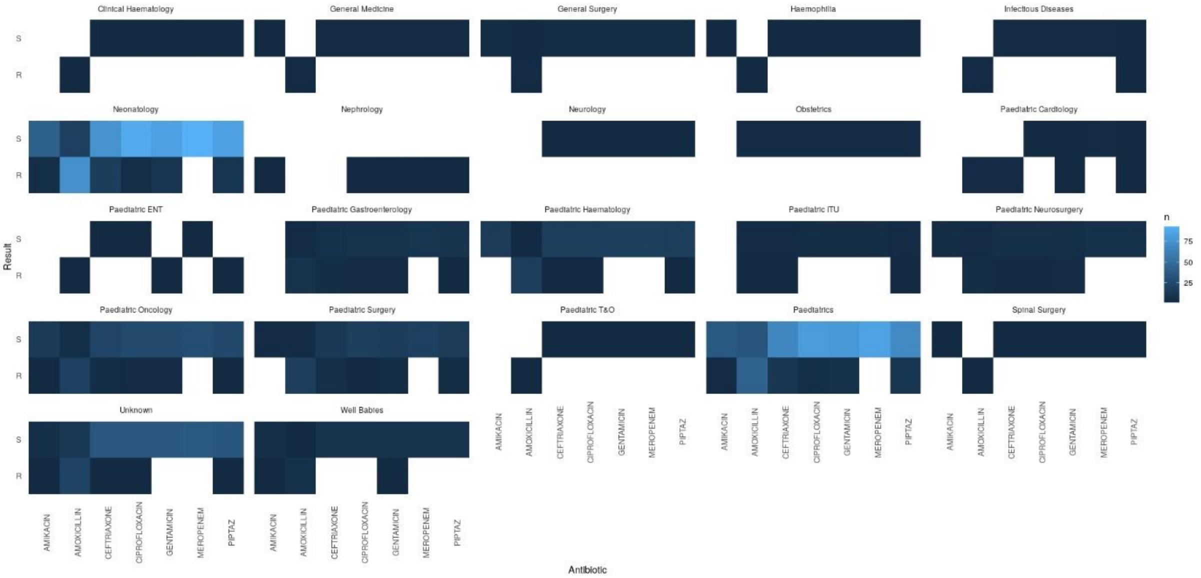

Figure S2: Phenotypic profile by primary treatment specialty. Colours represent the number of isolates resistant or sensitive to each antibiotic indicated.

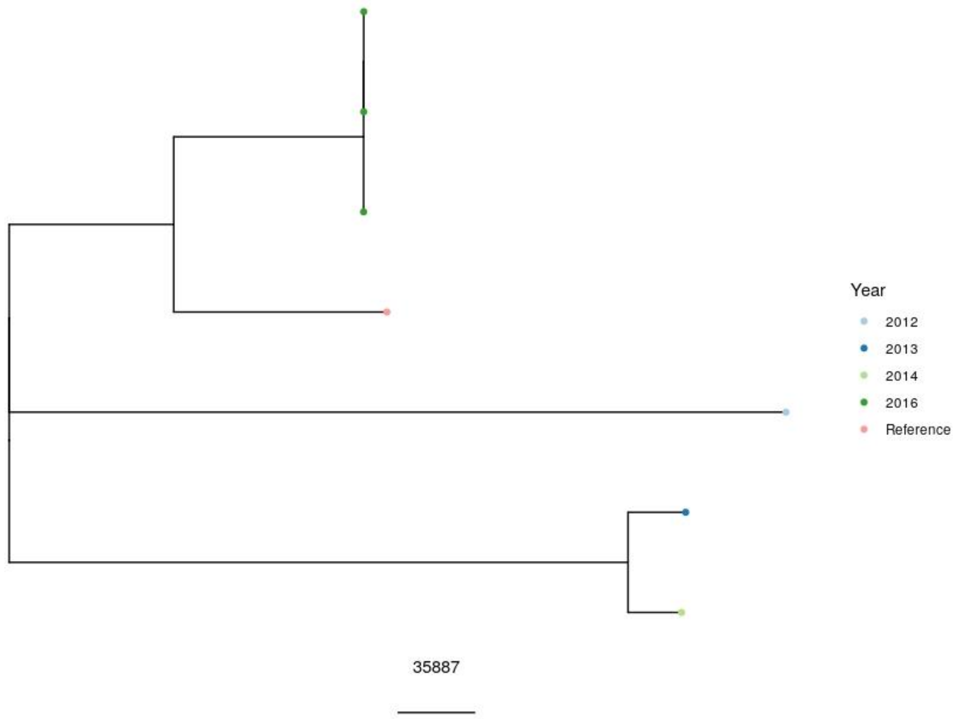

Figure S3 – Recombination corrected phylogenetic tree of the 6 sequenced *Serratia marcescens* isolates. The scale bar shows distances in SNPs.

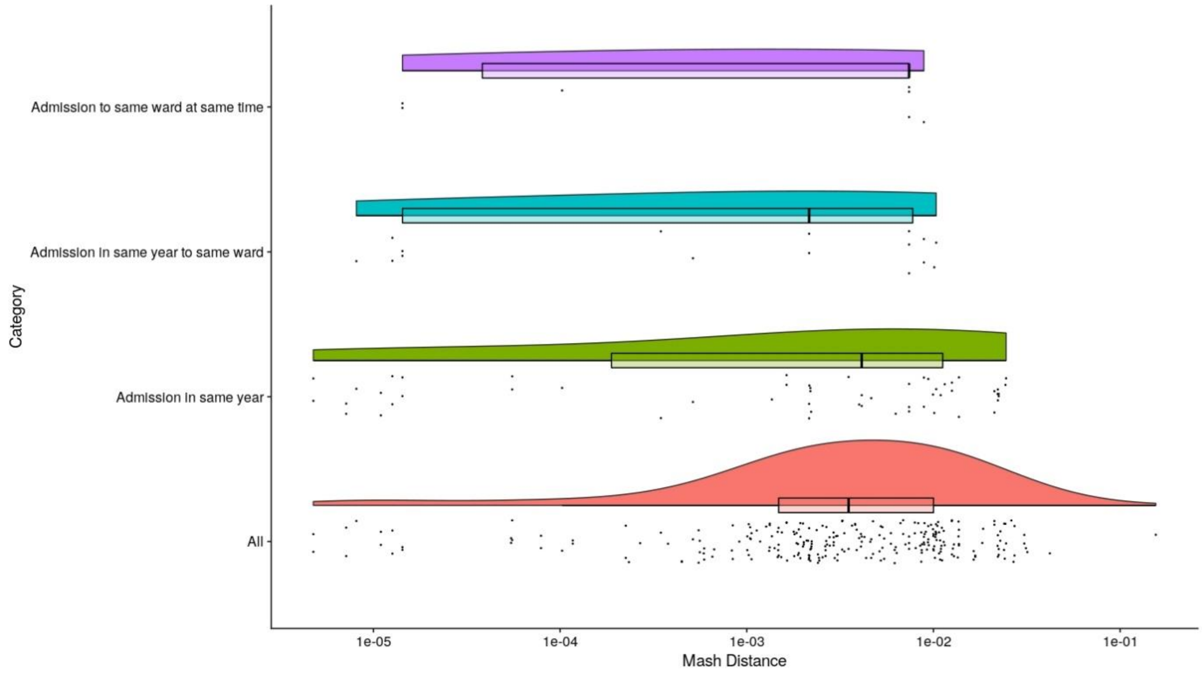

Figure S4 – rainbow plot showing distributions of Mash distances between isolates from patients with different degrees of epidemiological connections. The box plot shows median and interquartile range with overlying density plots to show the distribution of all datapoints. Number of datapoints

for each category – Admission to same ward at same time n = 7, Admission in same year to same ward n = 16, Admission in same year n=66, All n = 259.

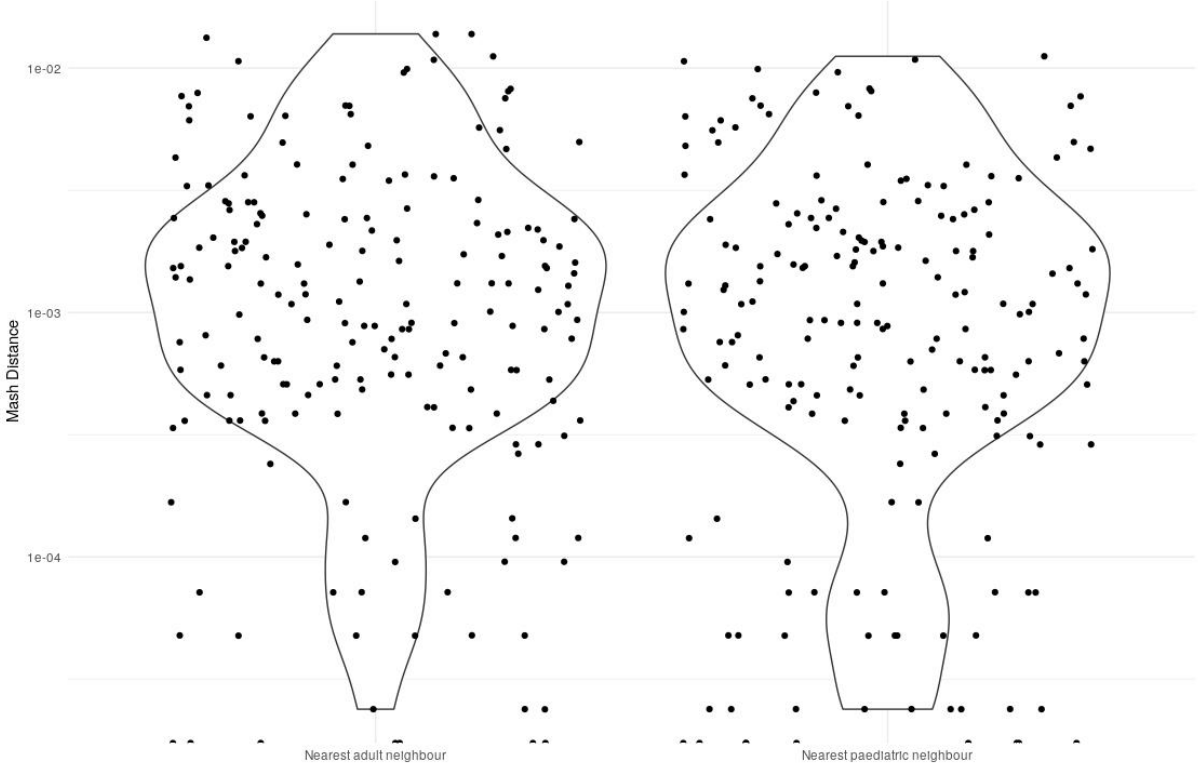

Figure S5: Distributions of Mash Distances for the distance to nearest adult neighbour (left) and nearest paediatric neighbour (right). One comparison per patient per group was selected for this analysis. There are n=193 comparisons for each category.
