# Supplementary material for: Molecular epidemiology and antimicrobial resistance phenotype of paediatric bloodstream infections caused by Gram-negative bacteria
Source: Commun Med (Lond). 2022 Aug 11;2:101. doi: 10.1038/s43856-022-00161-0 (PMC9372158; doi:10.1038/s43856-022-00161-0)
Supplement: Supplementary file 3 — Description of Additional Supplementary Files [file 43856_2022_161_MOESM3_ESM.pdf]

Description of Additional Supplementary Files

File Name: Supplementary Data 1

Description: Microbiological and patient characteristics of patients with Gram-negative bloodstream infections in Oxfordshire stratified by age of onset. P values represent row-wise comparisons using Fisher exact tests for categorical and Kruskal-Wallis tests for continuous variables.
